# The unintended consequences of combining equity measures with performance-based financing in Burkina Faso

**DOI:** 10.1186/s12939-018-0780-6

**Published:** 2018-09-24

**Authors:** Anne-Marie Turcotte-Tremblay, Manuela De Allegri, Idriss Ali Gali-Gali, Valéry Ridde

**Affiliations:** 1 University of Montreal Public Health Research Institute, 7101 Avenue du Parc, Room 3060, Montreal, QC H3N 1X9 Canada; 2 School of Public Health, University of Montreal, 7101 Avenue du Parc, Montreal, QC H3N 1X9 Canada; 3 Institute of Public Health, Medical Faculty, Heidelberg University, Im Neuenheimer Feld 130.3, 69120 Heidelberg, Germany; 4 Association Action Gouvernance Intégration Renforcement (AGIR), Ouagadougou, Burkina Faso; 5 Association Zama Forum pour la Diffusion des Connaissances et des Expériences Novatrices en Afrique (Zama Forum / ADCE-Afrique), Bobo-Dioulasso, Burkina Faso; 6 IRD (French Institute for Research on Sustainable Development), CEPED (IRD-Université Paris Descartes), Universités Paris Sorbonne Cités, ERL INSERM SAGESUD, Paris, France

**Keywords:** Performance-based financing, User fee exemption, Universal health coverage, Indigents, Unintended consequences, Burkina Faso, Multiple-case study

## Abstract

**Background:**

User fees and poor quality of care contribute to low use of healthcare services in Burkina Faso. The government implemented an innovative intervention that combines equity measures with performance-based financing (PBF). These health equity measures included a community-based selection of indigents to receive user fee exemptions and paying healthcare centres higher purchase prices for services provided to indigents. Research suggests complex interventions can trigger changes not targeted by program planners. To date, however, there is a knowledge gap regarding the unintended consequences that can emerge from combining PBF with health equity measures. Our objective is to document unintended consequences of the equity measures in this complex intervention.

**Methods:**

We developed a conceptual framework using the diffusion of innovations theory. For the design, we conducted a multiple case study. The cases were four healthcare facilities in one district. We collected data through 93 semi-structured interviews, informal discussions, observation, as well as intervention documents. We conducted thematic analysis using a hybrid deductive-inductive approach. We also used secondary data to describe the monthly evolution of services provided to indigent and non-indigent patients before and after indigent cards were distributed. Time series graphs were used to validate some results.

**Results:**

Local actors, including members of indigent selection committees and healthcare workers, re-invented elements of the PBF equity measures over which they had control to increase their relative advantage or to adapt to implementation challenges and context. Some individuals who did not meet the local conceptualization of indigents were selected to the detriment of others who did. Healthcare providers believed that distributing free medications led to financial difficulties and drug shortages, especially given the low purchase prices and long payment delays. Healthcare workers adopted measures to limit free services delivered to indigents, which led to conflicts between indigents and providers. Ultimately, selected indigents received uncertain and unequal coverage.

**Conclusions:**

The severity of unintended consequences undermined the effectiveness and equity of the intervention. If the intervention is prolonged and expanded, decision-makers and implementers will have to address these unintended consequences to reduce inequities in accessing care.

**Electronic supplementary material:**

The online version of this article (10.1186/s12939-018-0780-6) contains supplementary material, which is available to authorized users.

## Background

Achieving health equity remains a challenge in many low- and middle-income countries (LMICs). User fees significantly limit access to services, especially for the poor, while quality of care is often considered to be insufficient. In the pursuit of universal health coverage (UHC), governments are adopting a range of interventions to provide access to high-quality health services without exposing patients to financial hardship [1, 2]. Some approaches are primarily directed at service providers (supply side) to improve the quality of healthcare services, while others focus on beneficiaries (demand side) to reduce financial obstacles that limit access to care. Interventions that combine measures to improve equity in service use, quality of care, and financial protection may be promising, as they provide a more comprehensive response to health needs [1, 2].

In this vein, performance-based financing (PBF) is increasingly being adopted to improve the quantity and quality of healthcare services. However, few attempts have been made to combine PBF with equity measures that target vulnerable groups, in spite of emerging evidence suggesting PBF is not inherently pro-poor [3, 4]. In Cameroon, for example, a PBF program with specific measures to target the poorest found under-coverage was a concern. Indigents who attended the facility constituted only a tiny proportion of the population (maximum 0.7%) [5]. According to Renmans and colleagues [6], consensus exists on the fact that “*PBF is not adapted to tackle social determinants or health inequities*.” More broadly, it is possible that any purchasing mechanism, by being primarily focused on the supply side, has difficulty producing equity changes. Global health actors are consequently calling for strategic purchasing reforms such as PBF to be reoriented by linking them with additional measures that can promote equity and achieve universal health coverage by 2030 [7].

Innovating in this field, the government of Burkina Faso received financial and technical support from the World Bank to test PBF with different equity measures specifically targeting indigents [8]. Health equity measures included: a) a community-based selection of indigents, b) user fee exemption measures for indigents at point of service, and c) higher purchase prices to healthcare centres for some services delivered to indigents than for those provided to non-indigents. To select indigents, a local consultancy firm was contracted to adapt and reproduce the process described by Ridde, whereby village committees proposed lists of indigents that were then validated by the health centres’ management committees [9]. This method was chosen by the Ministry of Health based on evidence of its effectiveness [9, 10]. Committees of community representatives relied on their knowledge of the population and living conditions to select indigents based on locally accepted definitions: individuals who are extremely disadvantaged socially and economically, unable to look after themselves, and devoid of internal or external resources [9]. The definitions of indigence could be heterogeneous across communities because they were intended to be adapted to local realities. According to intervention reports, 15–20% of the population in the selected healthcare centres’ catchment areas were supposed to receive indigent cards to access free healthcare services and medication [11, 12].

For the PBF component of this intervention, healthcare centres were paid a unit purchase price for each targeted service delivered (e.g. curative consultation for adults). Healthcare centres that met quality-related performance targets following verifications were also eligible to receive bonus payments. Quality scores of over 50% were used to inflate PBF payments. PBF payments were used to fund expenditures, increase bank reserves, and pay bonuses to employees of the healthcare centres [13].

The intervention described above is complex, given the number of interacting components, the number of groups and organizational levels targeted, and the number outcomes [14, 15]. Many global health actors are concerned that implementing such a complex intervention could produce unintended consequences that are outside the targeted objectives of the intervention [16–18]. These unintended consequences are defined as changes for which there is no purposeful action or causation and that occur in a social system as a result of adopting, adapting, or rejecting an innovation such as PBF [19]. These changes can be desirable or undesirable, depending on the stakeholders’ perspectives. They can affect various actors, such as service users, providers, donors, community members, and government representatives.

To our knowledge, the intervention implemented in Burkina Faso presents a unique opportunity to develop scientific knowledge because no study has been conducted to date on the unintended consequences of combining PBF with equity measures for indigents in Africa. Although program planners believe these approaches may have a synergistic potential, the combination may not work out as planned. Interaction between the different rationales, goals, and operating procedures may produce unintended consequences. Thus, our objective is to document the unintended consequences of equity measures integrated into the complex PBF intervention in Burkina Faso.

## Methods

### Theoretical framework

This study was based on Rogers’ diffusion of innovations theory [19]; our aim was to focus on the intervention’s adoption and adaptation from a broad perspective, in order to capture unintended consequences. While the theory provides an original approach to the study of PBF in a low-income setting, it has also been used in the past to analyze the consequences of health innovations [20–22]. According to the theory, combining PBF with health equity measures constitutes an innovation because both practices are perceived as new by adopters. The theory stipulates that diffusion of innovations usually widens the socioeconomic gap. However, when special efforts are made by a diffusion agency, it is possible to narrow or at least not to widen it.

**Fig. 1:**
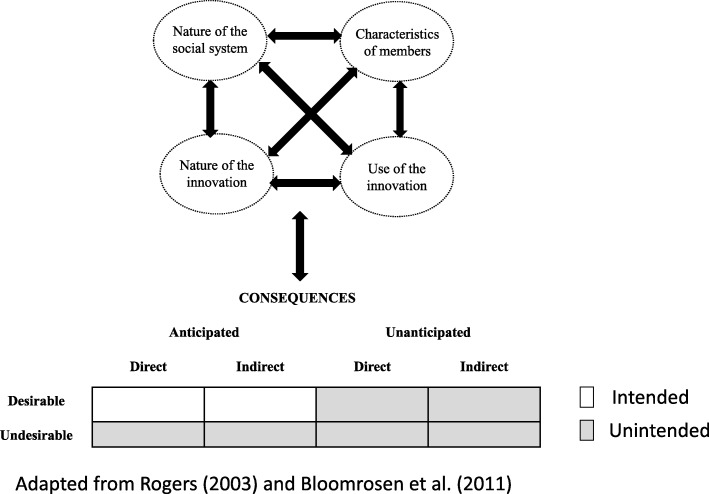
Conceptual model

To understand an innovation’s diffusion process and consequences, we can examine four main dimensions: 1) the characteristics of the members of the social system (e.g. their knowledge and beliefs about the intervention, attitude towards change); 2) the nature of the social system (e.g. norms, culture, characteristics of the organization); 3) the nature of the innovation (e.g. relative advantage, compatibility, triability, complexity); and 4) the use of the innovation (e.g. its re-invention) [19]. These dimensions can interact to influence the emergence of various types of consequences. Rogers classified consequences as: 1) desirable or undesirable, 2) direct or indirect, and 3) anticipated or unanticipated. To operationalize these concepts, we considered desirable consequences to be those that are functional (positive) for the social system and undesirable consequences to be those that are dysfunctional (negative). A consequence could potentially be both desirable and undesirable, depending on the point of reference [21]. We considered consequences as anticipated if they were explicitly or implicitly addressed in the implementation guides. In accordance with Ash et al.’s [21] approach, we considered direct consequences to be related to processes and indirect consequences, to outcomes. Like Bloomrosen et al. [20], we considered that intended consequences tend to be those that are simultaneously desirable and anticipated. In contrast, unintended consequences tend to be those that are undesirable and/or unanticipated. Our rationale for these assumptions is that program planners generally intend to make changes they consider desirable and that they can anticipate. We also assume program planners do not purposefully target changes they consider undesirable or have not anticipated. We have shown the applicability of this typology elsewhere [23]. Figure 1 illustrates our conceptual model [23].

### Study setting

Burkina Faso is a low-income country where health needs are a major concern. The maternal mortality ratio is 371/100,000 live births [24]. The under-5 mortality rate is 89/100,000 live births [25]. In the country’s National Health Development Plan for 2011–2020 [26], priority issues include: 1) poor performance of the health system, especially in terms of governance and service delivery; 2) lack of human resources; 3) inadequate quality and supply of health products such as medication and vaccines; 4) insufficient coverage and maintenance of infrastructure, equipment, and logistics; 5) poor health information systems management; and 6) inadequate funding for health and poor management of resources.

To address these issues, the government of Burkina Faso conducted a pre-pilot PBF test in 2011 in three districts [27]. In 2014, this intervention was modified to include the health equity measures described in the introduction. It was also expanded to an additional 12 districts. To conduct an impact evaluation, funded by the World Bank, four intervention modalities were implemented across 15 districts [8]: 1) PBF1: healthcare centres were paid fixed unit prices for activity indicators achieved; 2) PBF2: PBF1 coupled with a community-based selection of indigents to be exempted from user fees at point of service; services provided to indigents were purchased at a higher unit price than those provided to non-indigents to compensate healthcare centres for financial loss due to unpaid user fees; 3) PBF3: PBF2 with higher unit prices for services provided to indigents, to motivate healthcare workers to treat indigents and to better compensate healthcare centres for financial loss (see Additional file 1); and 4) PBF4: PBF1 linked with a community-based health insurance program and a community-based selection process for indigents. In this article, for reasons of feasibility, we focus on the PBF1 and PBF3 intervention modalities (see details in the section *Sampling of cases*).

The present study took place in a district of Burkina Faso where achieving equitable use of healthcare services remains a challenge. The district population was estimated at 135,740 in 2016, with more than 50% living in poverty [28]. Of the 19 primary healthcare centres in this district, five were allocated to PBF1, seven to PBF2, and seven to PBF3. Although PBF started in January 2014, cards to identify the selected indigents were only available for distribution in November 2015. Healthcare workers, however, were encouraged to begin applying user fee exemptions for indigents before then. Implementation guides describe the planned intervention model and the different actors supposed to be involved in the selection process [13, 29].

### Research strategy

This research was nested within a larger longitudinal process evaluation of the intervention [8]. For the design, we conducted a contrasted multiple case study with several embedded levels of analyses [30]. The cases were four primary healthcare centres, called *Centres de santé et de promotion sociale* (CSPS – centres for health and social promotion).

### Sampling of cases (facilities)

Case selection was done shortly after the intervention launch and followed a multistage screening procedure [30, 31]. First, we identified a district that represented the normal healthcare system context and was located in a relatively safe area for researchers. Within this district, we assessed the CSPSs’ levels of performance on key activity indicators for maternal and child health. We ranked the CSPSs into quintiles to select centres with contrasting levels of performance. We then asked key informants (i.e., members of the district management teams) in each district to help us select facilities that were representative of their performance category and that offered opportunities for significant insight [30, 32, 33]. This dialogue with local informants helped us avoid selecting cases that were outliers or unrepresentative. For this analysis specifically, we decided to focus on facilities in the first and third intervention arms only. We selected the first intervention arm (PBF1) because it represents a common PBF model that is being widely implemented in low-income countries, thus increasing the pertinence of the results. We selected the third intervention arm (PBF3) because it is an innovative PBF model with health equity measures. The final set of cases consisted of two high- and low-performing PBF3 facilities and two high- and low-performing PBF1 facilities. The data collected in the PBF3 facilities were primarily used to understand the implementation and various changes related to the equity measures integrated within the PBF intervention, while the data collected in the PBF1 facilities were primarily used for triangulation purposes and to better understand the overall context, while avoiding over-attributing relevance to the equity measures. We did not include PBF2 facilities, as the targeting intervention was comparable and only unit prices differed. We also excluded PBF4 facilities because the intervention model combining insurance with PBF is radically different and rarely used in other countries, thereby limiting the utility of results. Table 1 describes each facility included.

**Table 1:** Description of four cases

Descriptors	Facility 1	Facility 2	Facility 3	Facility 4
**Intervention arm**	PBF3	PBF3	PBF1	PBF1
**Initial performance**	Low	High	Low	High
**Type of facility**	CSPS, public, not-for-profit	CSPS, public, not-for-profit	CSPS, public, not-for-profit	CSPS, public, not-for-profit
**Healthcare workers**	1 head nurse	1 head nurse	1 head nurse	1 head nurse
	2 itinerant health workers* (IHW)	1 IHW	1 nurse	1 IHW
		1 auxiliary midwife	2 IHWs	1 auxiliary midwife
	1 auxiliary midwife		1 midwife	4 trainees (temporary)
	1 IHW volunteer		1 auxiliary midwife	
			3 trainees (temporary)	
**Support staff**	1 drug depot manager	1 drug depot manager	1 drug depot manager	1 drug depot manager
	1 guard	1 guard	1 guard	1 guard
	1 janitor	1 janitor	1 janitor	2 janitors
**Number of villages in catchment area**	5	8	22	6
**Population in catchment area**	~ 8000	~ 3600	~ 11,000	~ 3700
**Easy access to paved road**	No	No	Yes	Yes
**Ethnic majority**	Dagara	Lobi	Lobi	Birifor, Djan
**Number of indigents selected (coverage rate)**	829 (10.4%)	566 (15.7%)	0 (0%)	0 (0%)
**Economic activities**	Agriculture	Agriculture	Agriculture	Agriculture
	Livestock farming	Livestock farming	Livestock farming	Livestock farming
	Production of local alcohol	Production of local alcohol	Production of local alcohol	Production of local alcohol
**Distinctive features**	Gardening during dry period	High migration rate	High migration rate	

*Itinerant health workers are employees in charge of promoting health, hygiene, and vaccination, notably through household visits and community gatherings. In practice, they also deliver healthcare services due to the shortage of healthcare workers

### Data collection method

We collected qualitative data during two sequential phases, with the first informing the methods used for the second. For the first phase, the first author conducted 3 months of fieldwork between January and April 2016. The researcher’s immersion in the milieu provided a better understanding of the context and helped create a relationship of trust with stakeholders. We visited each healthcare facility for a two-week period to conduct semi-structured interviews, informal discussions, and non-participant observation. Participants included a wide range of stakeholders, such as indigents, non-indigents, members of indigent selection committees, representatives from the *Comité de gestion* (COGES – healthcare facility management committee), community-based health workers (CHWs), healthcare workers, and patients. Participants were purposefully selected based on their ability to provide relevant information and their accessibility. Then, following the snowball approach, some key informants referred us to other potential participants who could shed light on the intervention. Using these approaches, we followed the diversification principle to select participants with a variety of intrinsic characteristics, such as different indigent statuses, occupations, and genders [33]. For the interviews, we constructed guides that drew on previous questionnaires used for research on the diffusion of innovations [34, 35]. We systematically recorded field notes on observations and informal discussions in research diaries. Observation sites included healthcare facilities, villages, and other social settings (both public and private). The first author also participated in a six-day annual PBF review meeting at the national level to triangulate data regarding unintended consequences, better understand the different contexts, and assess the potential transferability of results to other facilities in intervention districts.

For the second phase, the third author conducted 20 days of fieldwork in May 2016 to deepen our assessment of the relations between community verifications and equity measures for indigents. He conducted semi-structured interviews, informal discussions, and non-participant observation in each of the four facilities. To provide complementary data, he conducted an additional interview in December 2016 with a key stakeholder involved in indigent selection. The same procedure was used to select participants as described above. He recorded field notes in research diaries.

In total, we conducted 93 semi-structured interviews and recorded 241 observation sessions in research diaries. Applying the principle of saturation, we stopped collecting data when interviews and observations no longer provided information that was sufficiently different to justify continuing. Research team members produced verbatim transcriptions of interview recordings. Table 2 provides a breakdown of the qualitative data collected and analyzed. It should also be noted that the last author has in-depth understanding of the context, having participated in workshops to define the intervention process for the equity measures and taken part in follow-up meetings on this topic.

**Table 2:** Summary of data collected

	Quantity
Non-participant observation	
Sessions reported in field notes	241
Interviews	
At facility level	
Healthcare providers	15
Other support staff (drug depot manager, janitor, security guard)	13
Volunteers & trainees	7
Community leaders (e.g., COGES, selection committees & community health workers)	23
Service users (e.g. patients, indigents)	18
At district level	
Administrative staff (e.g. manager, accountant, data collection agent/photographer)	4
Members of contractualization and verification agency	4
Members of local association conducting community verifications	7
At national level	
Representative from the *Programme d’appui au développement en santé* (PADS – program to support health development)	1
Representative from the *Service technique* – *financement basé sur les résultats* (ST-FBR – results-based financing – technical service)	1
Total semi-structured interviews	93

We also used secondary data on healthcare services delivery that are publicly available on the Ministry of Health’s PBF portal (www.fbrburkina.org). These longitudinal data are collected monthly in each healthcare centre for PBF verifications. Healthcare workers report the quantity of healthcare services delivered to indigent and non-indigent patients, based on the medical registers. Then PBF officers verify the reported data by manually recounting the quantity of services. They enter the data into an electronic platform. We used the data collected between October 2015 and September 2016, that is, before and after fee-exemption cards were distributed to indigents starting in November 2015. The main sample for the quantitative component consisted of the two facilities with equity measures (PBF3) included in the qualitative phase. To assess the transferability of the findings across the study district, however, we examined all seven facilities within the district that were assigned to the same intervention arm as the two selected for inclusion in the qualitative component (PBF3) and for which data were available. To assess the transferability of findings more widely, we also examined all 196 facilities in the intervention districts that belonged to intervention arms with similar measures for indigents (PBF2 and PBF3) and for which data were available.

### Data analyses

The primary unit of analysis was the healthcare facilities and their catchment areas. We combined deductive and inductive thematic analysis [36, 37]. We began by developing a template of themes based on our theoretical framework. Then we carefully read the transcripts and field notes to assign the raw data to the predefined themes. At the same time, we derived new themes that were not included in the initial template but that emerged from the data and were judged relevant to our research topic. In some cases, we narrowed down and provided more focus to the initially defined themes to enhance their applicability to the data. We used QDA Miner 4 to code and retrieve text segments.

We also used descriptive statistics to examine how the quantity of services provided to indigents evolved over time, compared to those provided to non-indigents. We used Excel to create graphs and conducted a visual analysis to highlight patterns that emerged over time [38]. This complementarity information was used to triangulate some of the findings.

To classify the various unintended consequences, we followed a procedure previously developed and applied [23]. During the data analysis, we classified the different types of consequences based on the definitions of anticipated/unanticipated, desirable/undesirable, and direct/indirect presented above. To determine whether a consequence was anticipated or unanticipated by program planners, we reviewed intervention documents (e.g. guides, midterm reports) to better understand the design of the intervention model and its implementation. The document review enabled us to compare the program planners’ intended processes and outcomes to what actually emerged in real life. The titles of the documents reviewed are available in the references [12, 13, 29, 39]. In addition, we classified consequences as desirable or undesirable depending on whether we considered these changes to be functional (positive) or dysfunctional (negative) for the social system. Lastly, we classified consequences as direct or indirect depending on whether we considered these changes to be related to processes or outcomes.

We used a cross-case synthesis to draw general conclusions [30]. Following a replication logic, we considered that results arising independently from more than one facility are more powerful than those coming from a single facility, and thus gave the former more importance in the results section [30].

## Results

The results showed that community-based selection of indigents for user fee exemptions within a PBF program led to unintended consequences. Table 3 summarizes the results.

**Table 3:**
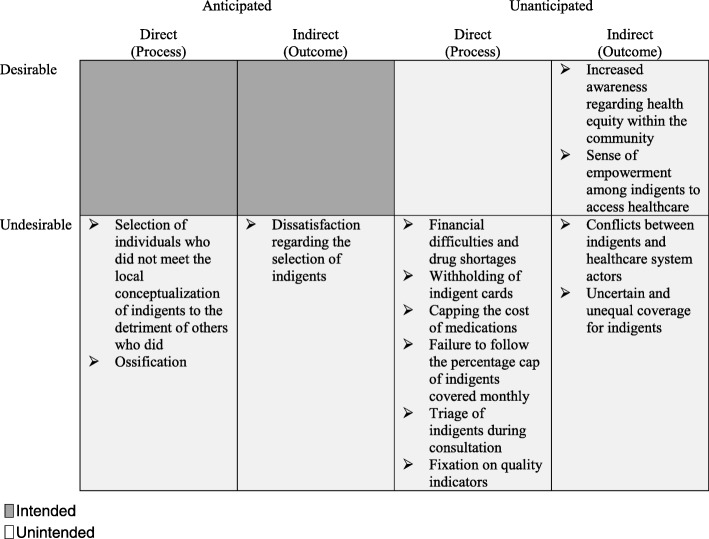
Classification of unintended consequences

Additional file 4 specifies how the anticipated consequences were explicitly addressed in the intervention guides

The subsections below describe in detail how interactions between the nature and use of the intervention’s different components (i.e., indigent selection, user fee exemption measures, and pro-poor purchase prices), the actors’ characteristics, and the nature of the social system led to the emergence of a wide array of unintended consequences.

### Selection of individuals who did not meet the local conceptualization of indigents to the detriment of others who did

Approximately 10 and 15% of the population were selected as indigents within the catchment areas of the two facilities in PBF3. Study participants and stakeholders who attended the annual PBF review meeting strongly affirmed that a portion of people selected as indigents did not meet the local conceptualization of indigents, that is, they were not individuals with no means to support themselves and not receiving assistance, such as widows, elders without children, handicapped persons, or orphans. Based on their knowledge of the communities and living conditions, some participants claimed that many individuals who were selected and obtained cards were not indigents. During interviews, some of these selected ‘indigents’ openly recognized that they did not truly qualify as such. These individuals received a card despite having income-generating activities, social support, ability to work, relatively high social status, belongings, etc. Examples of indigent card holders encountered during this study included the mother of the president of the COGES, a security guard and a janitor of a healthcare centre, a shop owner, a village chief, and a member of the village development committee. The latter benefited from the indigent card to the detriment of other unselected individuals who were considered worst-off.

*“They didn’t identify those who should have been…. Some people were selected, and others said [about them], ‘no, that person is working and has means!’”* (Community leader_50, interview, facility 2)

“*There are some indigents who do not have a card because it is not the real indigents who were selected.”* (Healthcare worker_16, interview, facility 1).

Numerous factors contributed to the selection process drift. First, study participants revealed that part of the selection was based on personal gain, affinity, social relations, and social status. For example, numerous CHWs and village councillors who sat on indigent selection committees obtained indigent cards for themselves or their immediate family members (see Additional file 2).

*“The treasurer [of the CSPS] is an indigent. Is that normal? …she works and has support. The old community health worker also has his indigent card. They wanted to pull a scam and play politics.”* (Healthcare worker cited in field notes, facility 2).

“*… this is a situation in which acquaintances and relations were used to distribute the indigent cards.*” (Healthcare worker_23, interview, facility 1)

Some CHWs in PBF3 facilities justified obtaining indigent cards for themselves or their family by arguing they had been doing volunteer work for the community without sufficient compensation. At the same time, some villagers and healthcare providers accused selection committee members of using the selection process to gain political influence for local elections. Others believed the high *relative advantage* of possessing a card played a role in the selection of individuals with questionable indigent status:

*“Because they’re saying everything will be free, everyone wants to be on the list.”* (Photographer for indigent cards_39, interview, across facilities)

Another important factor contributing to the selection of individuals not locally perceived as indigents was the confusion and misunderstanding regarding the number of indigents to select. After the selection of indigents had been completed, supervisors asked the committees to increase the numbers of indigents to reach a targeted number per village. As such, in both PBF3 facilities, a second selection was conducted, and people who did not meet the local conceptualization of indigents, including CHWs with revenues who sat on selection committees, were added to the lists.

*“We were identifying indigents and not reaching the [targeted] number. We were tired, and we just had to get it done…. [So] each one of us doing the selection decided to register himself….*” (CHW_15, interview, facility 1)

*“…they told us to stop because there were problems with the numbers in the register. We had to add, then we had to remove. At the same time, they told us to stop, so there were problems between the supervisors....*” (Photographer for indigent cards_39, interview, across facilities)

Moreover, the ‘photographers’—workers assigned to take indigents’ photos for the identification cards and record their GPS location—arrived unannounced in the villages to conduct their work. Not having been informed, some indigents had left the village with their families—for example, to cultivate, or to attend funerals—so the photographers were not able to take their photos. So, to reach the targeted number of indigents, the ‘photographers’ and CHWs in the first healthcare centre quickly replaced some of the absent indigents with other villagers available that day.

*“One day, we were all surprised to see the team with the photographer arrive in the village to take the pictures of the selected indigents. Because no one knew they were coming, some of the people selected as indigents were absent… I didn’t want to leave a void, so I simply replaced the people who were absent with others. When these people came back, they complained. I told them that I replaced them because they were not there and that it is not my fault because [the photographer] came without informing us in advance.”* (CHW_27, interview, facility 1)

*“The day of the selection, we went to his place and didn’t see him. So, we said [in the village], we need at least 200 people. So they had to just take whoever they found because the decision-makers were pressuring us.”* (Healthcare worker_16, interview, facility 1)

One ‘photographer’ reported that the remuneration modality, which was based on performance, also contributed to selecting individuals not on the initial list of indigents. The data collection agents were reportedly paid about 320 CFA francs (0.57 USD) for each indigent identified.

*“The clever ones, you’ll notice, started taking [photos of] all of the children who were at home to facilitate their work… It’s a strategy they made up.”* (Photographer_66, interview, across facilities)

Some selection committee members argued that the conditions under which the selection was conducted affected the quality of their work, especially due to the *complexity* of the task. Some participants noted, for example, that individuals doing the selection were not sufficiently trained, that the communication system was deficient, that not enough time was provided for the selection, and that they received no financial compensation for their hard work. Participants also revealed deficiencies within the committees involved in the selection process. For example, one CHW stated that he conducted the selection of indigents alone in his village. Meanwhile, in another centre, two members of the selection committee at the facility level revealed that they had not seen the final list of indigents, and one was unaware that indigent cards had been distributed in the catchment area during this study, claiming that *“the bureau didn’t do its job.”* Moreover, a midterm report [12] confirmed the committees that were initially supposed to be in charge of validating the lists of indigents (referred to as the local validation groups) were not implemented: *“…this structure was never created in the villages, given its relevance to realities on the ground. The main observation was that the community leaders held multiple responsibilities. Thus, the people who were part of the indigent selection committees were mostly the same people who were in the local validation groups”* (p. 15). Although these obstacles relate to the implementation process, they help explain the context in which gaming occurred for the selection of indigents.

Our observations and interviews suggested that selecting indigents based on personal affinity and personal gain was consistent with the broader social system and local stakeholders’ past experience. Study participants reported that relationships and informal networks are important for survival and prosperity, especially in a context of widespread poverty. They spoke often about the high rate of corruption within and outside the healthcare sector. As one participant described, malfeasance is not uncommon in new projects implemented by international organizations with limited funding and timeframes.

*“I see projects that come to the village, and the chief is asked to bring forward the indigents. Everyone gathers up their own family, even if they’re able to cover their own care.”* (Patient_10, interview, facility 1)

“*The country is corrupt! Here, everything depends on relationships.*” (Student midwife cited in field notes, across cases)

### Ossification

According to a midterm report [12], consideration was given to setting up a system to update indigent lists after the initial selection: *“This approach makes it possible to regularly update the list of indigent persons selected”* (p. 7). However, no update mechanism had been implemented at the time of this study. Thus, indigents who were absent when the photographer came to their village or people who fell into poverty after the selection were unable to obtain an indigent card. After the photos were taken, selection committees were unable to modify indigent lists. Many study participants did not know how long indigent cards were valid, and some believed changes would not be possible for the next 3 years. Indigent cards with identification errors could not be corrected, as they were manufactured in Vietnam. The selection process had a low level of *adaptability*, that is, stakeholders did not formally have the opportunity to make modifications according to their needs and constraints over time. Thus, the intervention led to a certain level of ossification, that is, organizational paralysis brought about by a rigid system and the presence of a centralized decision-making structure, as illustrated by the following citations:

*“Because they [decision-makers] say we can only review this in three years, we’ll go along with it to see what happens over the next three years and how they’ll select the indigents next time.... We’ll bear with it and keep advocating to see whether they can shorten that three-year period.”* (Healthcare worker_17, interview, facility 1)

*“If PBF [officials] don’t come back, how can we get that card for him? It’s a problem.”* (COGES_60, interview, facility 2)

*“We don’t know how we’ll get through this.”* (Head nurse cited in field notes, facility 1)

### Dissatisfaction regarding selection of indigents

In both facilities, study participants reported that the selection process led to frustrations, conflicts between actors, and a sense of injustice. Indigents omitted from the selection or absent when the photographer came demanded that the situation be rectified. Some individuals demanded to be selected as indigents due to the *relative advantage* of having free healthcare services, the perceived inequity of the selection process, and the lack of understanding regarding the definition of ‘indigents’. *“Why hasn’t anyone from my household been selected? Not a single person!? How is it that some benefit and others don’t?”* asked one member of the committee in charge of coordinating the selection at the facility level during a heated COGES meeting (facility 2). To appease these types of frustrations, selection committee members sometimes made false promises to the population, made apologies and distanced themselves from the selection process, arguing that it was the ‘community’ that chose the indigents.

*“…if I’d known, I wouldn’t even have gotten involved in this work. It caused us a lot of problems. In fact, every morning people would come to my house to ask whether a new list had opened up so they could register. This bothered me a lot. Also, it made me uncomfortable when some people scowled and got angry*.” (CHW_14, interview, facility 1)

“*People are envious. Some people want to really force their way into getting a spot, but it’s not for them.”* (COGES_59, interview, facility 2)

Despite these complaints, community members generally remained in favour of user fee exemptions for indigents.

“*In any case, the villagers said it’s a good project for the whole village.*” (CHW_27, interview, facility 1)

“*The people actually appreciated the idea of covering indigents. They even said that, if it were to really happen…then everyone will start to believe in ‘the white man’s paper’.”* (Volunteer IHW_11, interview, facility 1)

### Increased awareness regarding health equity within the community

The intervention triggered discussions and reflections within the community on health equity and the issue of indigence. For example, community members not selected as indigents engaged in discussions with healthcare workers and selection committee members to better understand the selection process and the reasons for their exclusion. This provided opportunities to explain the concept of indigence and the importance of providing access to services to the most vulnerable individuals.

*“In the community, some have welcomed it. Then there are others who say, no, if that’s how it is, then everyone is an indigent, even though they’re not indigents. So we explain often… it’s just to help the poorest…. Some understand, but others don’t.*” (Healthcare staff_17, interview, facility 1)

*“Some said, the entire village is made up of indigents, so we should select everyone. We said, no, it’s not like that. We explained to those people that there are selection criteria. We have to select the old widows who have no support, people with no support. Those are the people we chose.*” (CHW_27, interview, facility 1)

### Withholding of indigent cards

A major concern for study participants in the second facility was that some indigent cards were missing and never distributed to their owners. Healthcare workers and CHWs put the blame for these missing cards on the ‘photographers’ and technical difficulties with the equipment used to identify and photograph indigents (i.e., digital tablets). However, observation revealed that a head nurse—who did not approve of the selection of certain indigents and was concerned this process would negatively influence the medications stock—had surreptitiously removed some indigent cards before their distribution in the community. A district supervisor reported that this strategy had been used in other healthcare centres and recommended this approach to healthcare workers in the first facility to lower the number of indigents and limit the healthcare centres’ financial difficulties (as discussed in the next subsection).

*“Some head nurses filtered the cards, and when people ask for them, say they haven’t arrived. They say that every time. You just had to do the same thing.”* (Supervisor cited in field notes, facility 1)

### Financial difficulties and drug shortages

Healthcare centres in PBF3 received higher unit purchase prices for some targeted services provided to indigents (see Additional file 1). For example, in the first facility, a consultation for an indigent adult was purchased at 1020 F CFA (1.72 USD), and for a non-indigent adult, 140 F CFA (0.24 USD). In exchange for these subsidies, healthcare centres were required to provide free services and free medications to indigents. If the cost of the medication prescribed was higher than the lump sum provided through the unit purchase price, the COGES had to absorb the difference using their other sources of revenues (user fees and sales of medication to non-indigent patients). If the cost was lower, the COGES retained the profit.

*“A district supervisor said,* ‘*It’s not just the white man’s money. The COGES also has to contribute to the indigents’ medications.’ In response, the healthcare workers shook their heads in disapproval.”* (Field notes, facility 1)

Numerous participants, including healthcare workers and COGES members, argued that delays in PBF payments caused financial difficulties for healthcare centres and led to drug shortages. At the time of the study, these delays were more than 6 months for quantity-related payments and more than 1 year for quality-related payments. Participants complained that, without the revenues from medications provided to indigents, it was difficult to replenish the centres’ drug depots. Some participants feared this would lower the quality of care for patients, who would have to obtain their medications elsewhere.

*“We have to wait for PBF to come pay for the products the indigents used before placing another order. I find it a bit difficult.”* (Healthcare worker_51, interview, facility 2)

There was also consensus among healthcare workers and COGES members in the first healthcare centre that the unit purchase prices for services to indigent patients were insufficient to cover the cost of their medications and that the healthcare centres did not have enough *slack in resources* to ensure proper functioning of the user fee exemption for indigents. Participants believed the insufficiency of compensation was causing financial difficulties and could lead to drug shortages in the healthcare centre.

*“If we stubbornly continue to treat people with prescriptions costing up to 3,000 francs and the system only pays 800 francs, who loses in that case? It’s the COGES that will suffer, and over time, we risk not even having products here at the depot…. Ultimately the healthcare facility could be at risk for closure. People will prefer to consult where they can find the products.”* (Healthcare worker_11, interview, facility 1)

According to the intervention guide [13], the purchase prices were intended to “*encourage healthcare workers*” to provide services to the poor. In practice, however, the financial incentive was perceived as insufficient to trigger proactive strategies on their part. For many healthcare workers in the first facility (PBF3), the *relative advantage* of providing user fee exemptions to indigents was mitigated by the fact that the healthcare centre lost money when the value of the medication provided for free was higher than the unit purchase price. Consequently, no additional efforts or innovative strategies were deployed to provide more services to indigents specifically, as explained by this healthcare worker:

*“We didn’t think of doing that. When an indigent person comes in, we treat him, and that’s all…. We know that with this [intervention], sometimes we make money, and sometimes we lose.”* (Healthcare worker_17, interview, facility 1)

Healthcare workers from facilities without equity measures for indigents (PBF1) also expressed lack of support for intervention models that provide user fee exemptions for indigents, for fear that those caused financial difficulties.

### Multiple strategies adopted to limit services to indigents

**Fig. 2:**
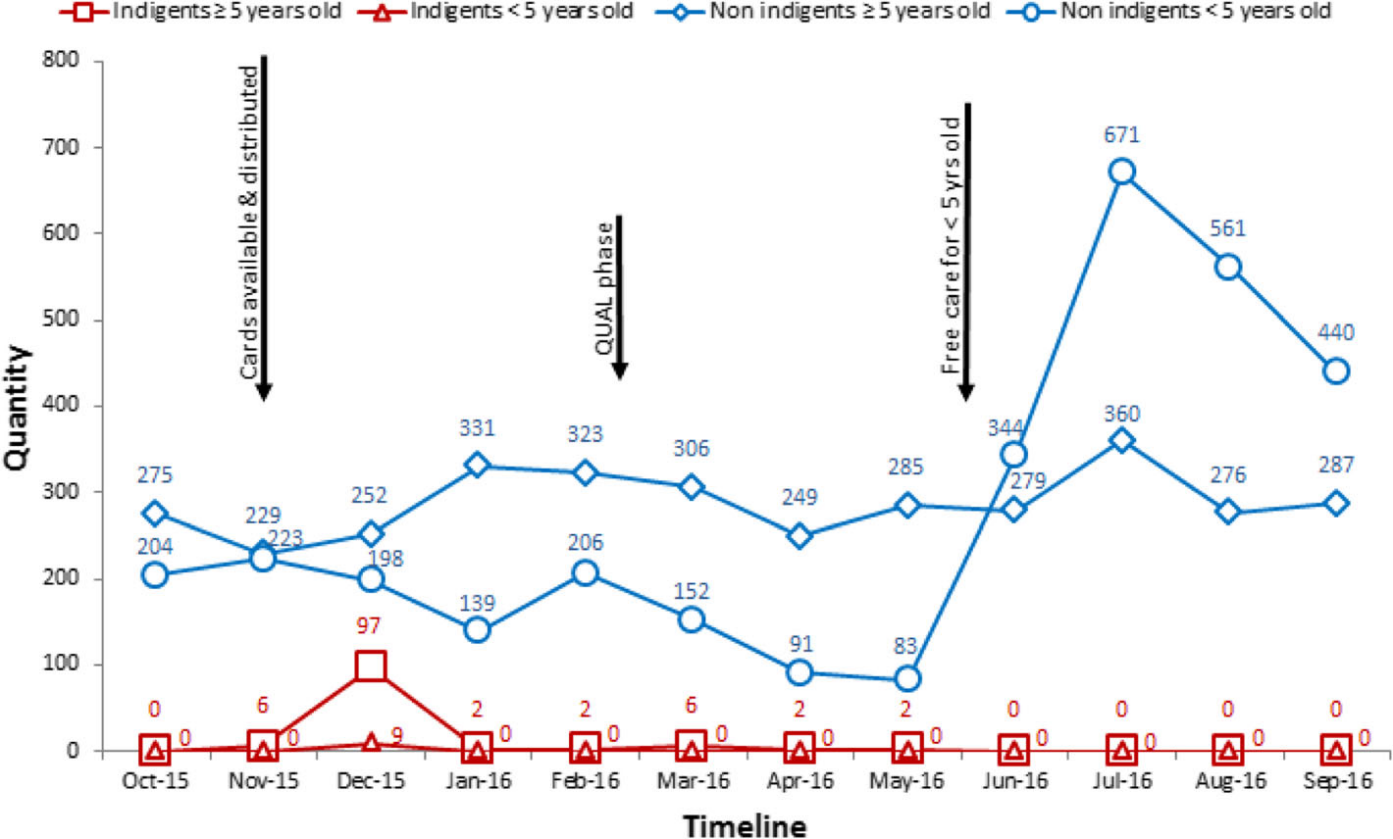
Facility 1 - Total number of new consultations for patients classified as indigents or non-indigents seen in curative care. Note: As shown by the qualitative data, the curves representing non-indigents are likely to include individuals who should have received user fee exemptions but were requested to pay, either because they did not possess an indigent card or because healthcare workers refused to recognize their indigent status. Similarly, the curves representing indigents may include individuals who received indigent cards even though they did not truly meet the local conceptualization of indigents

**Fig. 3:**
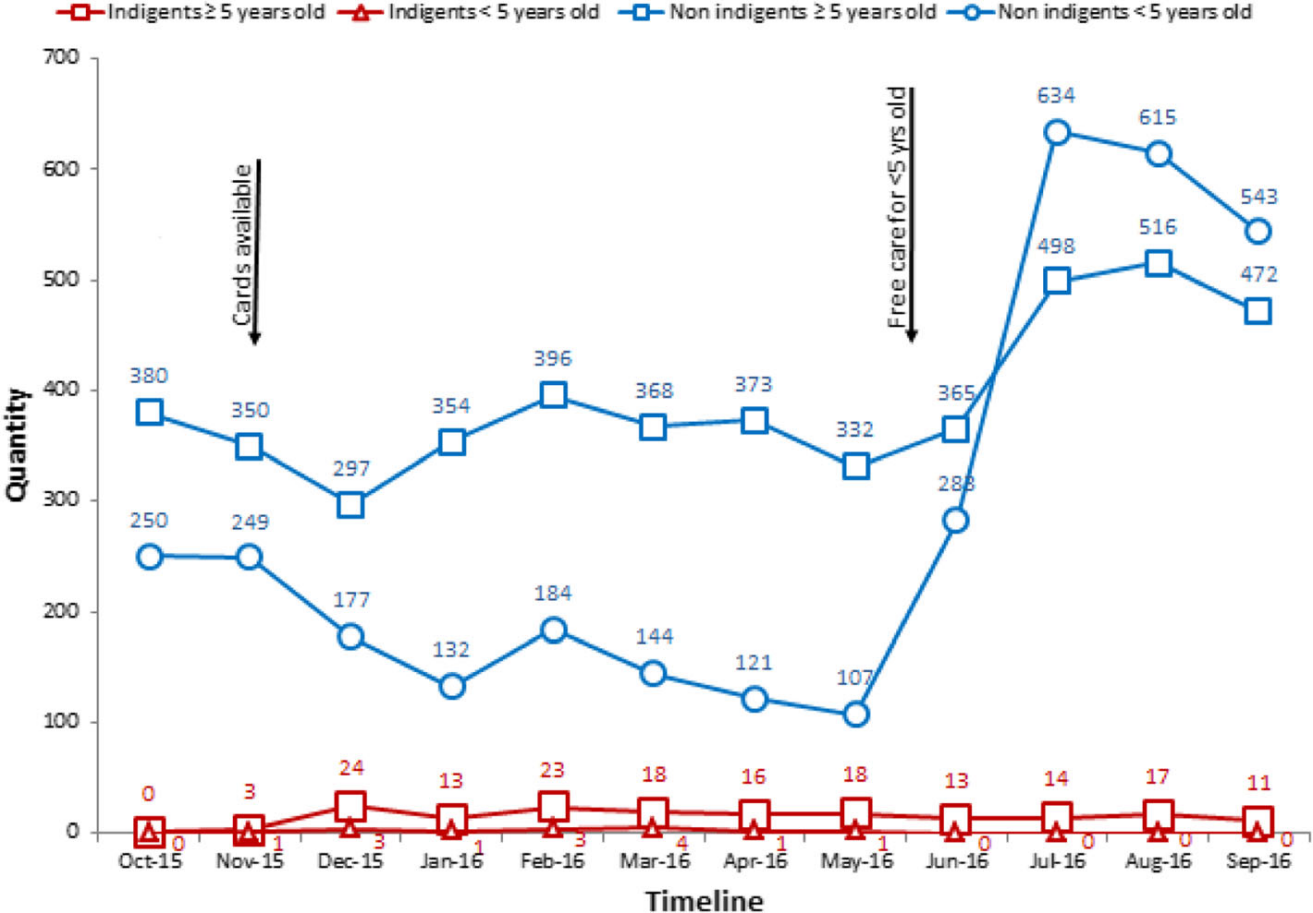
Facility 2 - Total number of new consultations for patients classified as indigents or non-indigents seen in curative care

**Fig. 4:**
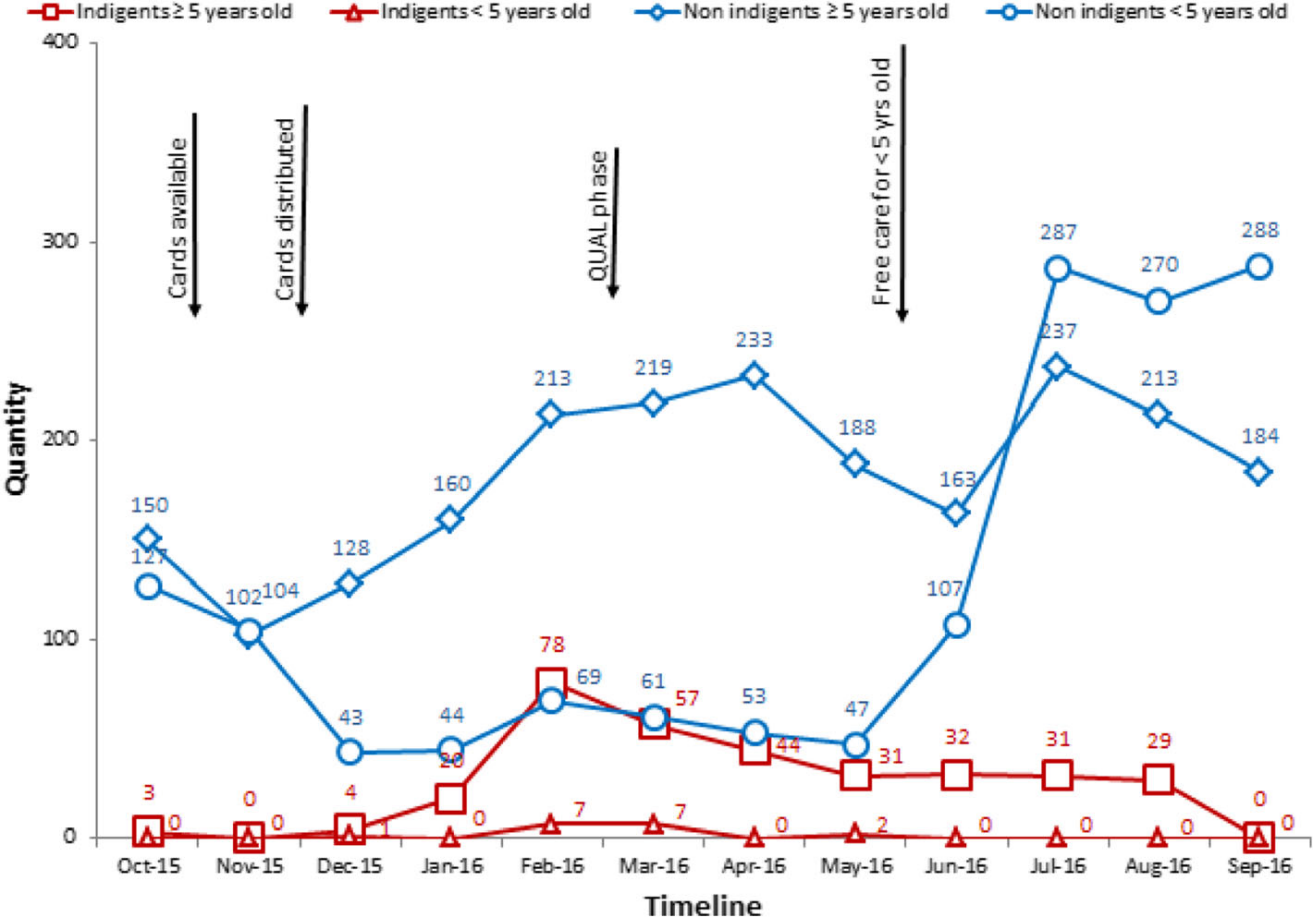
Average number of new consultations for patients classified as indigents or non-indigents seen in curative care in PBF3 facilities (*n* = 7) within the study district. Note: Healthcare centres (*n* = 7) for which the intervention arm was not specified in the database were excluded. PBF ceased to fund services delivered to indigent children under five once the free healthcare policy began in June 2016 because it covered their user fees and medications

Qualitative data showed that, shortly after the distribution of indigent cards, healthcare workers in the first facility (PBF3) adopted a series of strategic measures to limit the services and medications provided for free to individuals with indigent cards (as described in the subsections below). Secondary data on the quantity of services provided to indigent patients before and after indigent cards were distributed were consistent with these findings. Figures 2 and 3 show that, in both facilities with indigent targeting, the number of new consultations for patients classified as indigents increased considerably after indigent cards became available in November 2015. However, the following months saw rapid declines in the number of new consultations for patients classified as indigents. Since these declines are unlikely to have been due to sudden changes in morbidity prevalence or to the rapid cure of all indigents, these data support the findings that healthcare workers limited free services delivered to indigents. This is relatively consistent with the evolution of care in other healthcare centres belonging to the same intervention arm (PBF3) within the study district (Fig. 4), supporting the transferability of findings.

Moreover, the number of new consultations for patients classified as indigents did not follow seasonal patterns. Rainfalls generally influence the spread of diseases such as malaria, causing an increase in use of care between June and August. As expected from seasonal patterns, the number of new consultations for adult patients not classified as indigents peaked in July 2016. However, the number of new consultations for adult patients classified as indigents during this period actually followed the opposite pattern and gradually decreased or remained null. There is no reason to believe indigent adults would not be similarly affected by seasonal patterns. These secondary data also support the finding suggesting healthcare workers limited the delivery of free services to indigents. Within the study district (Fig. 4) and across intervention districts (Additional file 3), we also observed that the average number of new consultations for adult patients classified as indigents did not follow seasonal patterns.

The decline in new consultations for patients classified as indigents was more gradual for facility 2 than facility 1. This observation is consistent with the findings. In comparing the two facilities, we found that healthcare workers in facility 2 displayed a weaker understanding of PBF payment modalities for services and medications provided to indigents. They erroneously believed the PBF intervention would reimburse the entire cost of medications provided to indigents in addition to paying a unit purchase price for each consultation. Thus, at the time of the qualitative data collection, we found that, even though some indigent cards had been retained and there were delays in distributing cards (as shown in Fig. 3), healthcare workers in facility 2 delivered healthcare services to indigents. They expressed less disapproval of the indigent component of the intervention compared to workers in facility 1, whose disapproval was relatively high. Over time, however, the patterns in the number of new consultations for patients classified as indigents gradually moved in the same direction in both facilities.

It should be noted, however, that the peak in new consultations for non-indigent children coincided with the implementation of a new national policy for free healthcare to children under five in June 2016. At that time, the PBF intervention stopped purchasing services delivered to indigent children because children’s medications became covered by the national policy.

#### Capping the value of medication prescribed

To limit financial difficulties and protect the drug depot, healthcare workers and COGES members in facility 1 limited the prescribing of medications for indigents. They tried to keep the costs of those prescriptions under the PBF’s lump sum purchase prices. Interviews and examination of the indigent registry confirmed that prescriptions for indigents tended to cost around 1000 F CFA. Some supervisors and healthcare workers were concerned this practice was not rational and could have negative consequences on treatment effectiveness, healthcare system efficiency, and patients’ health.

*“If someone [indigent] comes in with simple malaria, we’ve been told we’re not supposed to exceed 850 F CFA for a prescription. ACTs for adults are 300 francs, the consultation is 200 francs, two paracetamol tablets are at least 150 francs. That’s 650 francs. At 850 francs, they say the healthcare centre makes a profit, but you’ve used gloves to examine the patient! Who covers the price of the gloves? What does the healthcare centre gain? Nothing! And, for example, if someone comes in with malaria plus pneumonia, whether you like it or not, the prescription costs more because you have to give an antibiotic, Amoxine, at least three tablets, plus Carbotoux [cough syrup], which goes for around 650 francs. Already that doubles or even triples their 850 francs. And if you don’t do that, the sick person will come back!… So they have to either increase the coverage or suspend their indigent business…. Now that they’ve imposed this on us, we’re obliged to do what they want.”* (Healthcare worker_16, interview, facility 1)

#### Failure to follow the percentage cap on indigents covered monthly

According to the intervention guidelines [13], free consultations for indigents should not constitute more than 10% of the total quantity of consultations to *“avoid the moral hazard”* (p. 53). Both the qualitative and quantitative data (see Figs. 2 and 3) showed this policy was not being systematically applied in either of the healthcare centres with user free exemptions. In facility 1, for example, 19% of curative consultations in December 2015 were provided to indigents. This proportion fell to under 1% in January 2016, when measures were taken to limit free consultations to indigents. Although healthcare workers in both centres knew about the percentage cap, there was misunderstanding regarding the correct percentage of patients that could be treated for free as indigents each month. Some participants also disagreed with applying a percentage cap.

*“At one point they [supervisors] had given us a monthly target rate. We exceeded it, and the indigents kept coming. We tried telling them we had to stop [for the month] and start again later, but they [indigents] didn’t accept that! They said I didn’t want to give the products for free.*” (Drug depot manager_22, interview, facility 1)

*“…if you reach the 40*^*th*^ *person, will you tell the others not to come?! Ah, no!”* (Healthcare worker_16, interview, facility 1)

#### Triage of indigents during consultations

Fearing financial difficulties due to the user fee exemption, the COGES of facility 1 requested that healthcare workers triage patients during consultations, then provide free services only to those they believed were ‘true’ indigents and require ‘false’ indigents to pay. The healthcare workers’ triage was based on their knowledge and perceptions of patients’ current socio-economic situation. COGES members and healthcare workers were confident they could accurately identify genuine indigents.

*“…we told them we would stop the system and verify for ourselves who the true indigents are. Currently, when an elderly person comes in and we see he doesn’t even have enough to pay for products, we qualify him as indigent. A blind person is an indigent, as is someone who lost their children and is alone without support. We take these people as indigents, and we make sure the prescription doesn’t exceed 800 francs*.” (Healthcare worker_11, interview, facility 1)

*“When healthcare workers take the cards from indigents, they ask them certain questions…. like, does he have anyone who can give him a hand and help him with his expenses? Questions like that*.*”* (COGES_14, interview, facility 1)

In contrast, a participant from facility 2 explained that they did not conduct any triage during the consultation because that would cause too much conflict with the local population, who traditionally are known to be warriors: *“the healthcare worker wouldn’t be able to work here anymore!”* This helps explain the more gradual decrease in the quantity of curative care to indigents in Fig. 3.

### Uncertain and unequal coverage for indigents

Both observation and interviews suggested that the selected indigents in facility 1 did not know in advance whether their healthcare would be free of charge. Upon consultation, some indigents had to decide whether to pay for the services they needed or leave without treatment. A number of factors influenced indigents’ access to free services, such as the healthcare workers’ triage of ‘true’ and ‘false’ indigents, the monthly percentage cap on indigents, the cap on the value of medications prescribed, sudden interruptions of the user fee exemption due to financial difficulties, indigents’ reactions to these measures, etc.

*“The first time, it was free, the second time it was free again, but the third time they told me to pay…. Ah, really, it discouraged me... If I don’t have money, I won’t come back [to the CSPS]. Now I know it’s not free.”* (Indigent patient_20, interview, facility 1)

*“There was an indigent man one time who went to the healthcare centre, and even though he was an indigent with a card, he paid a certain sum of money*.” (CHW_26, interview, facility 1)

### Fixation on PBF quality indicators

To promote orderliness, PBF evaluators deducted performance points if information in the registers was erased or crossed out. In both facilities with user fee exemptions for indigents, the staffs’ fixation on such performance indicators occasionally prevented indigents from receiving free care. For example, indigents who were accidentally listed in the wrong register were required to pay for services, as mistakes could not be erased or scratched out without risking losing PBF points.

*“It often happens that people have nicknames. If someone gives a name that isn’t on the indigent card, we’ll tell him he has to come back another day, because PBF doesn’t like it when we cross things out or erase things.”* (Drug depot manager_22, interview, facility 1)

*“When I arrived, I didn’t present the [indigent] card right away and they recorded my information in the register. After I showed them the card, they said I still had to pay for the medication, and I paid.”* (Indigent_18, interview, facility 1)

### Conflicts between indigents and health system actors regarding user fee exemptions

In facility 1, indigents expressed great dissatisfaction and lack of trust regarding healthcare workers and selection committee members because of the strategies used to limit their access to free healthcare and medications; indigents accused them of cheating and scamming. This experience was discouraging for some indigents.

*“They don’t understand why they were promised free healthcare services through these indigent cards and then later told they had to pay for these services. So they said it’s the healthcare workers who are playing politics on them…. many people came here to complain, saying that I had told them that with the card they would have full and free healthcare services and that the products prescribed to them would also be free, and yet that’s not the case at all.”* (COGES_14, interview, facility 1)

### Sense of empowerment for indigents to access healthcare

Many participants in both facilities argued that the user fee exemptions initially increased the selected indigents’ sense of empowerment to access healthcare services. It facilitated their decisions and actions to seek healthcare services more quickly. This finding was consistent with healthcare workers’ reports and the quantitative data indicating that the user fee exemption policy triggered a steep rise in attendance at health centres, at least until the services were curtailed.

*“It’s better because the [decision to seek] healthcare is in the hands of the indigent person. Under the previous system, the indigent was objectified. The person’s relatives decided everything.”* (COGES_59, interview, facility 2)

*“Their morale improved and they became brave…. All the old sick people who had been hiding came out.”* (COGES_21, interview, facility 1)

However, this initial sense of empowerment did not always translate into greater access to free healthcare services over time, due to the curtailing of services described above.

## Discussion

As postulated by the diffusion of innovations theory, we found that the nature and use of the intervention interacted with the social system and the characteristics of the different actors to trigger unintended consequences. One of the main findings was that different types of actors deliberately re-invented elements of the intervention over which they had control to strategically increase its relative advantage and cope with implementation challenges, thereby triggering unintended consequences. More specifically, many selection committee members partly re-invented the selection process to benefit personally from access to free healthcare services. In contrast, for many healthcare workers and COGES members, the relative advantage of providing free healthcare services and medications to indigents was insufficient due to the perceived low unit purchase prices for services to indigents (as conceived in the initial intervention model), the late payments (the implementation of the innovation), and the healthcare centre’s financial constraints (the nature of the local context). Healthcare workers deliberately modified the intervention model in different ways to make it more compatible with local resources and their own needs by retaining indigent cards, capping the value of medications provided, triaging patients into ‘true’ and ‘false’ indigent categories, etc. Although such re-invention was perceived as desirable by some local actors, it can also threaten the theoretical basis and equity implications of the intervention depending on the nature of the modifications to essential components [40].

### Application of the theory

Rogers suggests that one way to better understand the consequences of innovations is to classify them in a taxonomy [19]. Program evaluators and researchers tend to focus on certain types of consequences (e.g. desirable and anticipated) while neglecting others (e.g. undesirable and unanticipated) [41]. Thus, conceiving an inclusive typology *ex ante* compels stakeholders to consider the possibility that interventions can produce consequences that are not intended. In this study, we found Roger’s classification useful for conceptualizing different types of consequences, broadening our focus beyond intended consequences during data collection, and organizing the presentation of results.

One challenge we encountered, however, was in determining whether consequences were anticipated or unanticipated, since this could vary depending on the perspectives of the different types of stakeholders (e.g. researchers, policy makers, healthcare workers). As described in the theoretical framework, we classified consequences as anticipated if they were addressed in the intervention’s implementation guides (see Additional file 4). However, guides were sometimes unclear and imprecise regarding anticipated consequences outside the targeted objectives. Change agents hired to develop intervention guidelines do not always have a comprehensive understanding of the scientific knowledge and do not always openly disclose undesirable consequence that could undermine intervention models. Thus, while the concept of ‘anticipation’ was useful to guide our focus during data collection, its application was more problematic for a clear-cut classification of consequences.

### Targeting and user fee exemption policies

Our findings are consistent with past research suggesting that user fee exemption policies can lead to unintended consequences. With regard to indigent selection, for example, a study in Madagascar reported that village workers’ own interests influenced the selection and that individuals who were not selected complained [42]. Multiple studies have also found that user fee exemption policies can lead to reimbursement delays, revenue losses for health centres, or the unavailability of drugs [16, 43–46]. In line with our findings, studies in Mali, Senegal, and Madagascar found that healthcare workers adopted various strategies to reduce the scope of free care for targeted groups due to implementation dysfunctions, sometimes leading to complaints from the targeted population [42, 44, 46]. One study on targeting the poorest in a PBF program in Cameroon also found negative reactions among community members, such as jealousy [5]. The finding that user fee exemptions increase indigents’ sense of empowerment has also previously been documented [47].

Unlike in other studies, however, healthcare workers in Burkina Faso did not explicitly report feeling exploited or overworked with regard to providing services to indigents, although they did report an increase in the use of services [16]. This difference may be due to the limited number of indigents covered by the user fee exemption policy, the healthcare centres’ available capacity, the staff’s strategies to limit free services, and the timing of the data collection, as the use of services varies over the year.

### Combining PBF with equity measures

Innovation clusters, such as combining PBF with user fee exemption measures for indigents, may be useful to respond to the growing concern that PBF inadequately addresses inequity in access to care [7]. We found, however, that packaging PBF with user fee exemption measures for indigents can lead to unintended consequences. First, the healthcare workers’ fixation on PBF quality indicators sometimes hindered access to free healthcare services for indigents. Second, the low purchase prices for services were perceived as insufficient to motivate providers to target indigents and to cover the costs of the medication. A similar result was found in Cameroon, where some healthcare workers complained that the costs of treating indigents within a PBF program often surpassed the amount received [5].

However, we do not have quantitative data to confirm or challenge the participants’ claims that services to indigents were in fact costing more than the unit purchase price. Caution is advised when interpreting this result, as healthcare workers may have ulterior motives. Past studies have reported that user fee exemption policies may result in a loss of income for healthcare workers [16]. Thus, in the present study, it is unclear whether such practices might have influenced the healthcare workers’ negative discourse regarding the purchase prices for services to indigents. Those prices were supposed to be higher than simple cost recovery, to motivate healthcare workers to actively seek out the very poor, but as these services were not costed before the intervention was implemented, prices were set arbitrarily. The healthcare workers’ views and beliefs regarding the intervention were important because they translated into concrete actions that affected the quality and adequacy of services delivered to indigents. This highlights the pivotal role of “street-level bureaucrats”, who have a wide scope of discretion when implementing policies [48, 49]. As was found in this study, capping the value of medications given to indigents can lead to ineffective treatments and exacerbation of diseases, although a proper audit would be useful to evaluate the threat to quality of care. Program planners need to calculate adequately the real costs of treating indigents with medications to ensure they are not putting healthcare workers in a conflict of interests, where they have to choose between providing needed medications to indigents and protecting their own or the healthcare centre’s financial interests [42]. The importance of adequately calculating fixed purchase prices for user fee exemption policies in Burkina Faso has been highlighted in the past [50].

### Policy implications

The results of this study have policy implications, as governments in LMICs and funders search for strategies to promote the human right to health and to achieve universal health coverage [51]. Global health authorities have affirmed that it would be an unacceptable trade-off to *“first include in the universal coverage scheme only those with the ability to pay and not include informal workers and the poor, even if such an approach would be easier”* [52]. Thus, even if PBF funding ceases, global health actors must consider intervention models that can reach those most in need. In Burkina Faso, for example, a law on universal health insurance (n° 060–2015/CNT) stipulates that the state is the debtor for the indigents’ subscriptions (article 48) [53]. In this context, this study’s results may help decision-makers appreciate the implementation challenges and unintended consequences that can emerge from a community-based selection of indigents. The results are also pertinent with respect to the implementation of the country’s national health financing strategy for universal health coverage (2016–2013), which established strategic purchasing as one of its pillars [54].

Combining user fee exemption policies with PBF is likely to continue to be met with criticism and resistance from local healthcare workers if program planners do not resolve implementation challenges such as lack of starting funds, long reimbursement delays, or insufficient incentives. Past experiences suggest that, for a financing policy to be implemented successfully, budgets must be realistic and lost revenues need to be replaced in a timely manner to ensure a smooth flow of resources [55]. As McPake et al. [56] argue, quick action without sufficient preparation could lead to a deterioration in service quality. The practical issues of UHC implementation need more attention and research [1].

### Limitations of the study

Despite our rigorous design, this study does present potential limitations. First, some participants may have tried to portray the intervention positively, either to attract more international aid or due to fears of loss of confidentiality. However, this would have led to an underestimation of undesirable consequences rather than an overestimation. The high number of participants and the researcher’s immersion in the milieu reduced the risk of such potential biases. Second, while long observation periods within a few healthcare centres increased the credibility of results, they may have limited the degree to which findings may be generalized to other contexts or settings. It is possible that the implementation process differed between districts and intervention modalities, challenging transferability of results. However, we triangulated the results from our study facilities with multiple sources of qualitative and quantitative data based on broader samples (e.g. routine data, discussions during a one-week national PBF meeting, intervention documents). Moreover, we worked with local management teams to carefully select healthcare centres that were considered representative of the normal context. A third limitation is that we did not conduct observations during the training of local actors and the indigent selection process. This may have introduced potential biases in data collection (e.g. memory bias) and may have resulted in our capturing only a portion of the unintended consequences. Finally, we found that dealing with language differences was a challenge. In interviews, some participants spoke in their second language (French), while others spoke in their native language and relied on an interpreter. While this may have limited some participants’ ability to express themselves, we do not believe it affected the validity of results, given the large number of participants and the triangulation of data.

### Directions for future research

This study suggests numerous paths for future research. It would be interesting to use quantitative methods to conduct complementary statistical analyses. This could enable us, for example, to: 1) compare the value of medications prescribed to indigent vs. non-indigent patients; 2) assess the number of false inclusions and exclusions on indigent rolls; and 3) assess the cost-effectiveness of pro-poor targeting in comparison to other health equity measures. It would also be interesting to examine how the leadership and management of the intervention at the national and district levels influenced the implementation challenges that emerged. Studies have found that management and leadership practices, including personal initiatives of district leaders, effective supervision, and commitment of the district health management team and local government officials, are critical for successful implementation of exemption policies and UHC reforms [1, 57, 58].

## Conclusions

In the pursuit of universal health coverage, international organizations and governments of LMICs are increasingly considering strategies to combine PBF with health equity measures. Using the diffusion of innovations theory, we found that implementing PBF combined with user fee exemptions for indigents led to considerable unintended consequences in Burkina Faso. These unintended consequences can significantly undermine the overall effectiveness and equity of the intervention. To promote successful implementation, program planners need to ensure that local actors, such as healthcare workers, truly adhere to user fee exemption policies. This requires calculating the real costs of treating indigents with medications so that purchase prices paid to healthcare centres are adequate. Moreover, when combining PBF with equity measures, program planners should ensure that healthcare workers’ fixation on performance indicators does not undermine free services to indigents. Future research and evaluation of promising health interventions should focus well beyond intended consequences to consider unintended changes that may be less discernible but equally important.

## Additional files

Additional file 1: Examples of unit costs for healthcare services provided to indigents and non-indigents paid through the intervention (PBF3). (DOCX 16 kb)

Additional file 2: A member of the selection committee with indigent cards for his children. (DOCX 171 kb)

Additional file 3: Average number of new consultations for patients classified as indigents or non-indigents seen in curative care for healthcare centres in PBF2 and PBF3 across eight districts. (DOCX 149 kb)

Additional file 4: Explanations for the classification of anticipated vs. unanticipated consequences. (DOCX 161 kb)

## Abbreviations

CHW: Community-based health workers
COGES: *Comité de gestion* (healthcare facility management committee)
CSPS: *Centre de santé et de promotion sociale* (centre for health and social promotion)
F CFA: Franc from the *Communauté Financière d’Afrique*
GPS: Global positioning system
LMICs: Low- and middle-income countries
PBF: Performance-based financing
UHC: Universal health coverage
USD: United States dollar
