# Supplementary material for: The unintended consequences of combining equity measures with performance-based financing in Burkina Faso
Source: Int J Equity Health. 2018 Sep 24;17:109. doi: 10.1186/s12939-018-0780-6 (PMC6151907; doi:10.1186/s12939-018-0780-6)
Supplement: Supplementary file 1 — Examples of unit costs for healthcare services provided to indigents and non-indigents paid through the intervention (PBF3). (DOCX 16 kb) [file 12939_2018_780_MOESM1_ESM.docx]

**Additional File 1. Examples of unit costs for healthcare services provided to indigents and non-indigents paid through the intervention (PBF3)**

|  |  | Unit price  (F CFA) | |
| --- | --- | --- | --- |
|  | **Indicators** | **Non-indigents*** | **Indigents** |
| 1 | Number of new consultants of 5 years and older seen in curative care | 140 | 1020 |
| 2 | Number of new consultants of less than 5 years seen in curative care | 210 | 1270 |
| 3 | Number of days of patients placed under observation | 350 | 1350 |
| 4 | Number of counter-referrals received | 1400 | 1400 |
| 5 | Number of children completely vaccinated | 420 | 420 |
| 6 | Number of pregnant women who received the tetanus toxoid vaccine 2 or more times during the month | 350 | 350 |
| 7 | Number of prenatal consultations conducted in the health facility during the month | 560 | 560 |
| 8 | Number of postnatal consultations conducted in the health facility during the month (Day6–Day8 and Week6–Week8) | 700 | 700 |
| 9 | Number of births conducted with partographs during the month | 2100 | 3230 |
| 10 | Number of women (old and new) seen during the month for family planning and using oral or injectable contraceptives | 700 | 1330 |
| 11 | Number of women (old and new) seen during the month for family planning and using long-term contraceptive planning methods (intrauterine device & implant) | 1400 | 5150 |
| 12 | Number of new registrants aged 0-11 months seen in consultation for healthy infants | 140 | 140 |
| 13 | Number of children aged 12–23 months seen in consultation for healthy infants | 350 | 350 |
| 14 | Number of children aged 6–59 months being treated for moderate acute malnutrition (MAM) | 420 | 420 |
| 15 | Number of children aged 6–59 months being treated for severe acute malnutrition (SAM) without complication | 1050 | 1050 |
| 16 | Number of integrated household visits conducted | 4200 | 4200 |
| 17 | Number of people who underwent voluntary HIV testing (outside of people tested in the context of the prevention of mother-to-child transmission of HIV (PMTCT) | 700 | 700 |
| 18 | Number of pregnant women and partners who benefited from HIV testing in the context of the PMTCT | 700 | 700 |
| 19 | Number of HIV+ mothers who benefited from antiretroviral therapy | 3500 | 3500 |
| 20 | Number of children born from HIV+ mothers followed | 4200 | 4200 |
| 21 | Number of people living with HIV under ARV followed | 1400 | 1400 |
| 22 | Number of cases of smear-positive pulmonary tuberculosis (new cases and relapses) tested | 8400 | 8400 |
| 23 | Number of tuberculosis cases (all kinds) treated and declared cured or completed treatment | 11900 | 11900 |
| *Non-indigent patients also pay user fees in addition to these unit costs paid through PBF. A curative consultation costs 200 F CFA while being in observation costs a flat fee of 500 F CFA. | | | |
