# Supplementary material for: The unintended consequences of combining equity measures with performance-based financing in Burkina Faso
Source: Int J Equity Health. 2018 Sep 24;17:109. doi: 10.1186/s12939-018-0780-6 (PMC6151907; doi:10.1186/s12939-018-0780-6)
Supplement: Supplementary file 2 — A member of the selection committee with indigent cards for his children. (DOCX 171 kb) [file 12939_2018_780_MOESM2_ESM.docx]

**Additional File 3. A member of the selection committee with indigent cards for his children
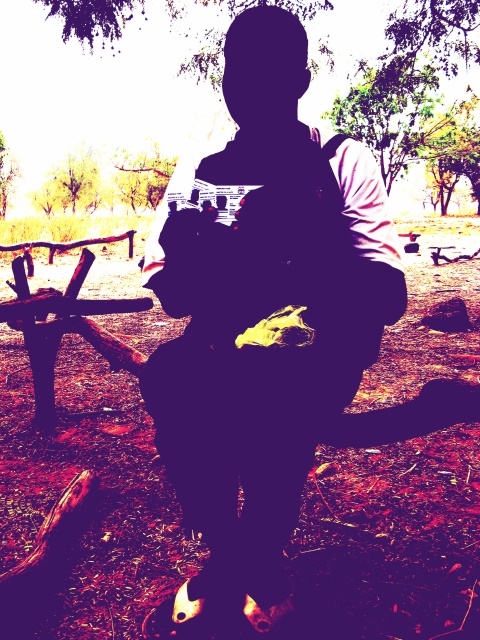
**

Description: A community health worker, member of the indigent selection committee, showing the indigent cards he obtained for himself and his immediate family members.
