# Supplementary material for: The unintended consequences of combining equity measures with performance-based financing in Burkina Faso
Source: Int J Equity Health. 2018 Sep 24;17:109. doi: 10.1186/s12939-018-0780-6 (PMC6151907; doi:10.1186/s12939-018-0780-6)
Supplement: Supplementary file 3 — Average number of new consultations for patients classified as indigents or non-indigents seen in curative care for healthcare centres in PBF2 and PBF3 across eight districts. (DOCX 149 kb) [file 12939_2018_780_MOESM3_ESM.docx]

**Additional File 4. Average number of new consultations for patients classified as indigents or non-indigents seen in curative care for healthcare centres in PBF2 and PBF3 across eight districts**


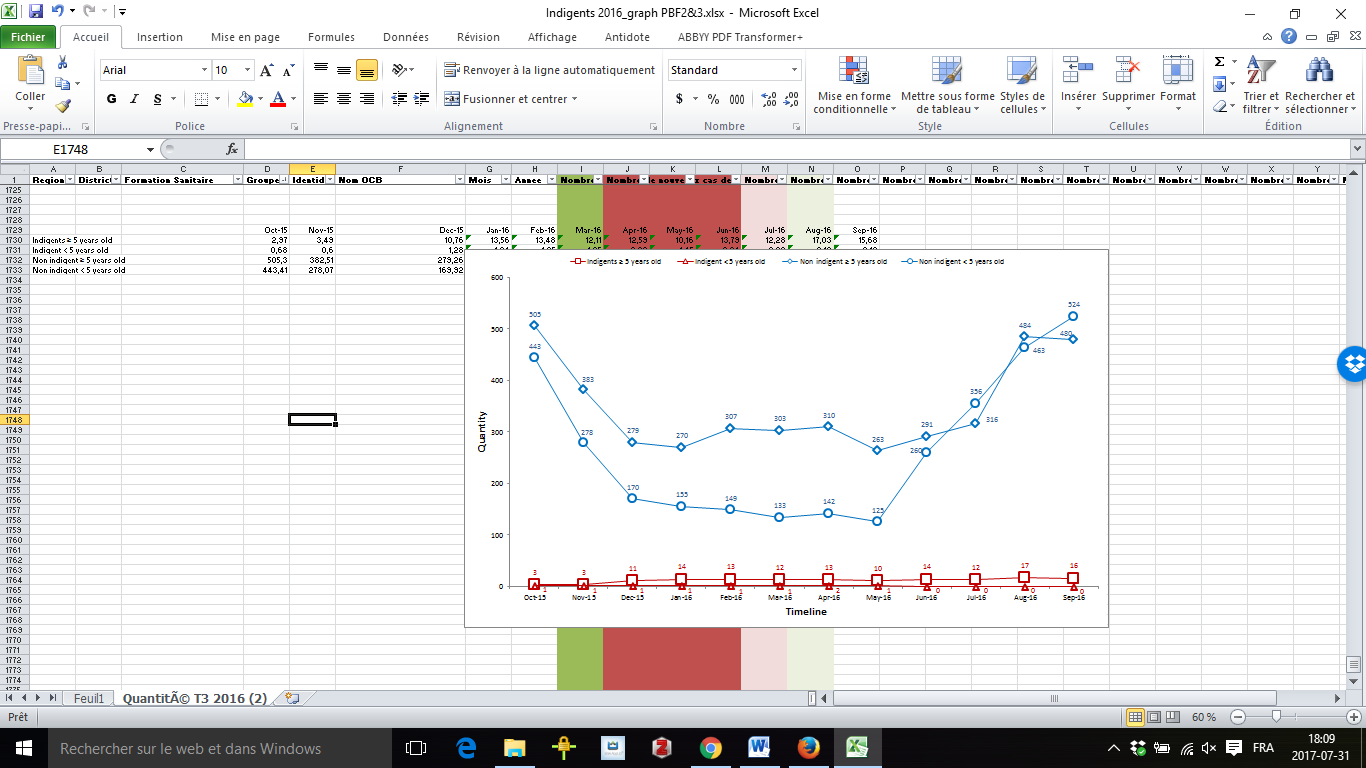
Note: Only data from healthcare centres belonging to the intervention arms with user fee exemptions for indigents (PBF2 & PBF3) were used for this graph to facilitate comparison. Districts with intervention arms combining PBF with mutual health organizations (PBF4) or who did not have user fee exemptions for indigents were excluded (PBF1).
