# Supplementary material for: The unintended consequences of combining equity measures with performance-based financing in Burkina Faso
Source: Int J Equity Health. 2018 Sep 24;17:109. doi: 10.1186/s12939-018-0780-6 (PMC6151907; doi:10.1186/s12939-018-0780-6)
Supplement: Supplementary file 4 — Explanations for the classification of anticipated vs. unanticipated consequences. (DOCX 161 kb) [file 12939_2018_780_MOESM4_ESM.docx]

**Additional File 2. Explanations for the classification of anticipated vs. unanticipated consequences**

|  | Anticipated consequences | | Unanticipated consequences | |
| --- | --- | --- | --- | --- |
|  | Direct (Process) | Indirect (Outcome) | Direct (Process) | Indirect (Outcome) |
| Desirable |  |  |  | Increased awareness regarding health equity within the community   - Not addressed in guides   Sense of empowerment among indigents to access healthcare   - Not addressed in guides |
| Undesirable | Selection of individuals who did not meet the local conceptualization of indigents to the detriment of others who did *“External validation was organized.”* (1)   - *“Strategies need to be developed to minimize targeting errors as much as possible.”* (2)   Ossification   - *“This approach makes it possible to regularly update the list of selected indigents”* (2) - *“The notion of indigence… raised some concerns regarding certain cases known in the society, particularly cases of temporary disability.”* (2) - *“There was some question about whether any process had been put in place to update the lists, to be able to take into account people who became indigent after the selection.”* (2) | Dissatisfaction regarding the selection of indigents   - *“The majority of people supported it, but not institutional actors such as health workers, or from other sectors, for whom financial management processes are routinely seen as opportunities to be exploited.”* (2) | Financial difficulties and drug shortages   - Not addressed in guides   Withholding of indigent cards   - Not addressed in guides   Capping medication costs   - Not addressed in guides   Failure to respect the monthly percentage cap of indigents covered   - Not addressed in guides   Triage of indigents during consultation   - Not addressed in guides   Fixation on quality indicators   - Not addressed in guides | Conflicts between indigents and healthcare system actors regarding user fee exemptions   - Not addressed in guides   Uncertain and unequal coverage for indigents   - Not addressed in guides   Risk of stigmatization of indigents   - Discussed during training sessions but not addressed in guides |
| 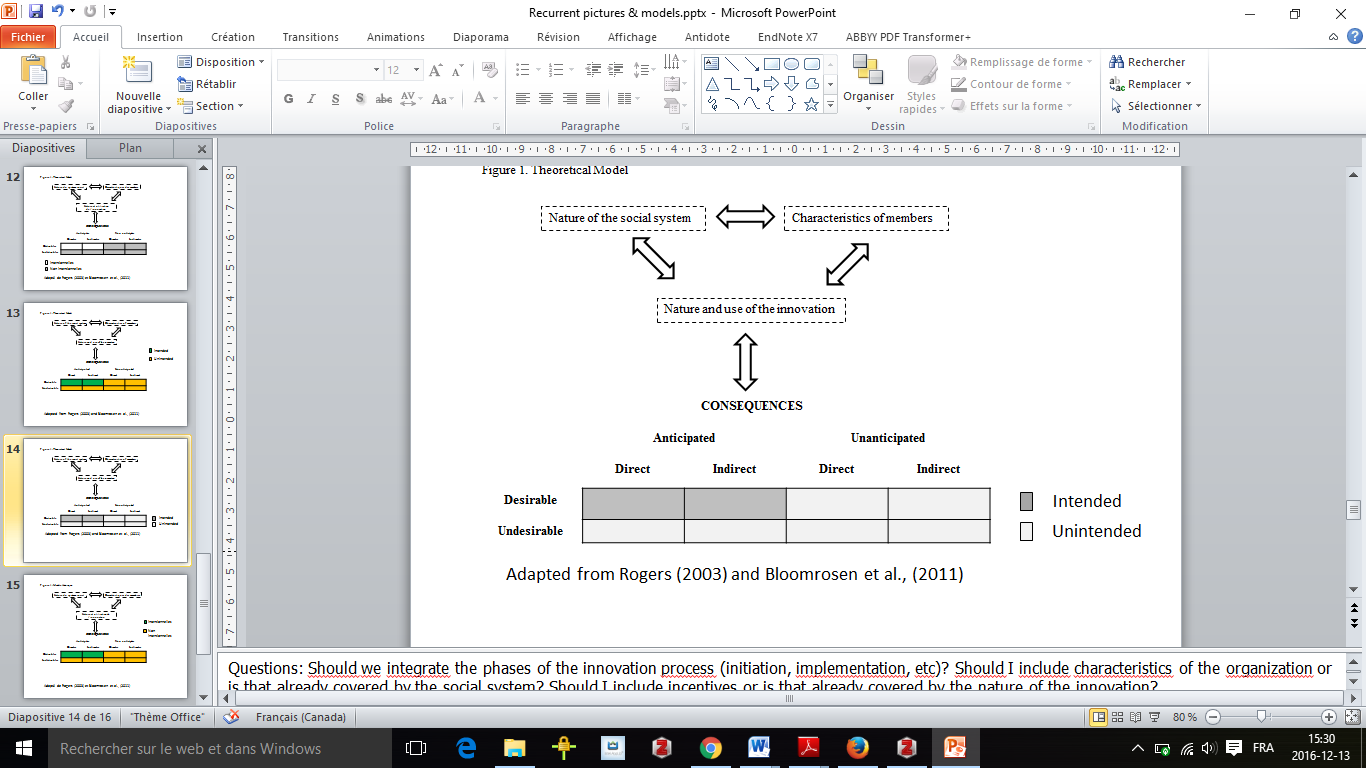 | | | | |

References

1. SERSAP. Stratégie de mise en place des CSI/GVL et de sélection des indigents. Ouagadougou.
2. Société d’études et de recherche en santé publique. Rapport d’étape de progrès de la mise en oeuvre et du suivi du processus communautaire de sélection des indigents au Burkina Faso. 2014.
3. Ministère de la Santé. Note d’information et d’orientation sur la prise en charge des indigents dans le cadre de la mise en oeuvre du processus de financement basé sur les résultats dans le secteur de la santé. 2014.
